# Transformation of CO_2_ and H_2_ to C_2+_ chemicals and fuels

**DOI:** 10.1093/nsr/nwad160

**Published:** 2023-05-30

**Authors:** Qingli Qian, Buxing Han

**Affiliations:** Beijing National Laboratory for Molecular Sciences, CAS Key Laboratory of Colloid, Interface and Chemical Thermodynamics, CAS Research/Education Center for Excellence in Molecular Sciences, Center for Carbon Neutral Chemistry, Institute of Chemistry, Chinese Academy of Sciences, China; Physical Science Laboratory, Huairou National Comprehensive Science Center, China; Beijing National Laboratory for Molecular Sciences, CAS Key Laboratory of Colloid, Interface and Chemical Thermodynamics, CAS Research/Education Center for Excellence in Molecular Sciences, Center for Carbon Neutral Chemistry, Institute of Chemistry, Chinese Academy of Sciences, China; Physical Science Laboratory, Huairou National Comprehensive Science Center, China; School of Chemistry and Chemical Engineering, University of Chinese Academy of Sciences, China; Shanghai Key Laboratory of Green Chemistry and Chemical Processes, School of Chemistry and Molecular Engineering, East China Normal University, China

## Abstract

This perspective highlights the progress of CO_2_ hydrogenation to multicarbon (C_2+_) products, by discussing some typical related works, future opportunities and challenges.

CO_2_ is a greenhouse gas, and is also a cheap, easily available and renewable carbon resource. Fixation of CO_2_ into chemicals has been extensively investigated in the forms of thermocatalysis, electrocatalysis and photocatalysis. CO_2_ is the highest oxidation state of carbon, and reduction of CO_2_ is usually required in its chemical utilization. Green hydrogen is an ideal reductant to transform CO_2_ into basic chemicals, because it can be obtained by splitting water with renewable electricity from solar energy. CO_2_ hydrogenation is among the most important areas of CO_2_ conversion, and considerable progress has been achieved in recent decades, especially in the production of C1 molecules such as CO, methanol, methane, and formic acid and its derivatives. In many cases, multicarbon (C_2+_) products are very useful in society. For example, acetic acid is a bulk and basic chemical, and its production capacity is ∼20 million tons/year. Ethanol and higher alcohols, as well as liquid (C_5+_) hydrocarbons, are not only basic chemicals but also engine fuels and fuel additives. Without doubt, the synthesis of C_2+_ chemicals using CO_2_ and H_2_ is of great importance. However, this is a challenging task because it involves controlled hydrogenation with simultaneous C–C bond formation. For a long time, there were only sporadic reports of the production of C_2+_ products from CO_2_ and H_2_. With the ever-increasing attention of society on CO_2_ utilization, reports on this topic have increased remarkably in recent years [1]. The progress can be briefly summarized according to product categories (Fig. 1). In this perspective, we highlight the progress in this area by discussing some typical related works.

**Figure 1.**
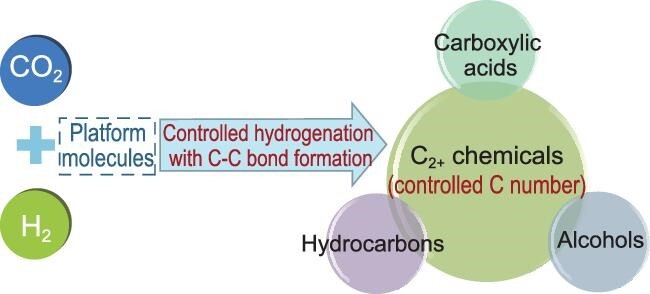
Synthesis of C_2+_ chemicals from CO_2_ and H_2_.

A route for acetic acid production via methanol hydrocarboxylation with CO_2_ and H_2_ was developed in 2016, where Ru_3_(CO)_12_-Rh_2_(OAc)_4_ was used as a catalyst [2]. The mechanistic study verified that CO_2_ directly took part in the reaction, which is different from the well-known methanol carbonylation pathway. There were also several breakthroughs in synthesizing higher carboxylic acids from CO_2_ and H_2_. A pioneering work showing higher carboxylic acid synthesis from olefins, CO_2_ and H_2_ at 180^o^C was reported in 2013. This was accelerated by a catalytic system consisting of [RhCl(CO)_2_]_2_ catalyst, PPh_3_ ligand, CH_3_I promoter and acidic additive (p-TsOH·H_2_O) [3]. In this work, CO was generated *in situ* via a reverse water gas shift (RWGS) reaction, which reacted with the olefin via carbonylation to produce carboxylic acids. In 2019, a strategy of carboxylic acid production from ethers, CO_2_ and H_2_ was put forward. This was catalyzed by IrI_4_ catalyst with LiI promoter at 170°C [4]. The catalytic system simultaneously transformed ether and CO_2_ into olefin and CO respectively, which further formed the carboxylic acids.

In 2016, a Pt/Co_3_O_4_ catalyst was reported to accelerate the synthesis of C_2+_ alcohols via CO_2_ hydrogenation, with a space time yield (STY) of 0.509 mmol g_cat_^−1^ h^−1^ for alcohols and a C_2+_OH selectivity of 82.5% at 200^o^C [5]. Later, it was reported that ethanol may be generated via CO_2_ hydrogenation in water solvent over CoAlO_x_ catalyst with a selectivity of 92.1% and activity of 0.444 mmol g_cat_^−1^ h^−1^ at 140°C [6].

CO_2_ hydrogenation to liquid hydrocarbons is usually promoted by heterogeneous catalysts, which may simultaneously catalyze the RWGS reaction and the subsequent Fischer–Tropsch synthesis (FTS). The RWGS reaction is endothermic and a higher temperature is needed to produce enough CO, whereas the FTS reaction is exothermic and higher temperatures tend to inhibit the formation of larger hydrocarbons. To overcome this thermodynamic limitation, an additional zeolite catalyst may be appended to reform the smaller hydrocarbons into larger ones. A highly efficient Na–Fe_3_O_4_/HZSM-5 catalyst operating at 320^o^C was designed according to this method, where gasoline fuel (C_5_–C_11_) composed of aromatics, naphthenes, isoparaffins, olefins and n-paraffins was produced [7]. Homogeneous catalysis is known for its high efficiency at a lower temperature. At 180°C, high efficiency was realized by coupling the homogeneous RuCl_3_ catalyzed RWGS reaction and heterogeneous Ru^0^ catalyzed FTS reaction to generate C_5_–C_28_ n-paraffins in a batch reactor [8]. Beyond CO_2_ hydrogenation, this study opens up the possibility of combining homogeneous and heterogeneous catalysts to achieve eminent cascade reactivity in a single reactor. The CO_2_ may also be firstly hydrogenated to methanol and subsequently reformed as liquid hydrocarbons in zeolite, and at 340^o^C the bifunctional In_2_O_3_/HZSM-5 catalyst demonstrated excellent performance when synthesizing gasoline (C_5_–C_11_) consisting of isoparaffins, n-paraffins, olefins and aromatics [9]. Direct hydrogenation of CO_2_ into C_5_–C_26_ n-paraffins and minor olefins was also realized over a simple Co_6_/MnO_x_ nanocatalyst at 200^o^C in a batch reactor [10]. No CO or methanol was detected in the reaction, and CO_2_ was directly reduced to CH_2_/CH_3_ species and transformed into liquid hydrocarbons on the catalyst surface.

There are still plenty of opportunities and challenges in this promising area. Firstly, some new reactions are to be developed. For example, the conversion of CO_2_ and H_2_ to basic chemicals such as C_2+_ aldehydes, diols and polyols, dicarboxylic acids and dioxane, has rarely been reported. The synthesis of C_2+_ carboxylic acids from CO_2_ and H_2_ currently needs a certain organic substrate, while highly efficient synthesis of the desired acids by direct conversion of CO_2_ and H_2_ has rarely been reported. Besides the new reaction routes, novel, cheaper and more efficient catalysts are to be designed and fabricated. The key to discovering new reactions is the development of novel catalysts that may engender new reaction pathways. In many instances, noble metal catalysts are used, especially in homogeneously catalyzed CO_2_ hydrogenation to C_2+_ products. Development of sustainable metal catalysis or organocatalysis is of great importance, but is still a challenge. The reaction courses of CO_2_ hydrogenation to C_2+_ chemicals are usually complex and the conditions are relatively harsh, which makes the mechanistic study more difficult. Well-designed experiments and advanced apparatus are needed to get more microscopic and *in situ* data on the reaction process, and theoretical calculations could also help produce convincing explanations. The rational catalyst design, on the basis of mechanistic understanding at the molecular level, would greatly expedite progress in this area.
